# Implications of anemia in patients undergoing PCI with Impella-support: insights from the PROTECT III study

**DOI:** 10.3389/fcvm.2024.1429900

**Published:** 2024-07-18

**Authors:** Batla Falah, Björn Redfors, Duzhi Zhao, Aditya S. Bharadwaj, Mir Babar Basir, Julia B. Thompson, Rajan A. G. Patel, Michael J. Schonning, Arsalan Abu-Much, Yiran Zhang, Wayne B. Batchelor, Cindy L. Grines, William W. O’Neill

**Affiliations:** ^1^ Clinical Trials Center, Cardiovascular Research Foundation, New York, NY, United States; ^2^ Division of Cardiology, NewYork-Presbyterian Hospital/Columbia University Medical Center, New York, NY, United States; ^3^ Department of Cardiology, Sahlgrenska University Hospital, Gothenburg, Sweden; ^4^ Division of Cardiology, Loma Linda University Medical Center, Loma Linda, CA, United States; ^5^ Center for Structural Heart Disease, Department of Cardiology, Henry Ford Health Care System, Detroit, MI, United States; ^6^ Ochsner Medical Center, New Orleans, LA, United States; ^7^ Inova Center of Outcomes Research, Inova Heart and Vascular Institute, Inova Fairfax Medical Campus, Falls Church, VA, United States; ^8^ Northside Hospital Cardiovascular Institute, Atlanta, GA, United States

**Keywords:** high-risk percutaneous coronary intervention, anemia, dialysis, major adverse cardiovascular and cerebrovascular event, bleeding

## Abstract

**Background:**

Anemia is prevalent among patients with cardiovascular disease and is associated with adverse outcomes. However, data regarding the impact of anemia in high-risk percutaneous coronary intervention (HRPCI) are limited.

**Objectives:**

This study aimed to evaluate the impact of anemia in patients undergoing Impella-supported HRPCI in the PROTECT III study.

**Methods:**

Patients undergoing Impella-supported HRPCI in the multicenter PROTECT III study were assessed for anemia based on baseline hemoglobin levels according to World Health Organization criteria. Patients were stratified into three groups, namely, no anemia, mild anemia, and moderate or severe anemia. Major adverse cardiovascular and cerebrovascular events (MACCE: all-cause death, myocardial infarction, stroke/transient ischemic attack, and repeat revascularization) at 30 and 90 days, and major bleeding events were compared across groups.

**Results:**

Of 1,071 patients with baseline hemoglobin data, 37.9% had no anemia, 43.4% had mild anemia, and 18.7% had moderate or severe anemia. Anemic patients were older and more likely to have comorbidities. Anemia was associated with higher MACCE rates at 30 days (moderate to severe, 12.3%; mild, 9.8%; no anemia, 5.4%; *p* = 0.02) and at 90 days (moderate to severe, 18.7%; mild, 14.6%; none, 8.3%; *p* = 0.004). These differences persisted after adjustment for potential confounders at 30 and 90 days, and sensitivity analysis excluding dialysis showed similar results. Major bleeding at 30 days was also higher in anemic patients (5.5% vs. 1.2%, *p* = 0.002).

**Conclusion:**

Baseline anemia in Impella-supported HRPCI is common and independently associated with MACCE and major bleeding, emphasizing its significance as a prognostic factor**.** Specific management strategies to reduce anemia-associated MACCE risk after HRPCI should be examined.

**Clinical Trial Information**

Trial Name: The Global cVAD Study (cVAD)

ClinicalTrial.gov Identifier: NCT04136392

URL: https://clinicaltrials.gov/ct2/show/NCT04136392?term=cvad&draw=2&rank=2

## Introduction

Anemia is a widely prevalent hematologic condition affecting 24.3% of the world population across all ages (1). It increases with age, reaching a prevalence of 39%–51% among patients aged over 65 years old (2). In patients undergoing percutaneous coronary intervention (PCI), the prevalence of anemia has been reported to be between 10% and 46.4% (2–8). Hemoglobin concentration (Hgb) values serve as the commonly used measurements for diagnosing anemia. The reference Hgb values for defining anemia are largely based on the definition provided by the World Health Organization (WHO) in 1968 (9). The presence of underlying anemia is linked with poor outcomes in cardiac patients including coronary artery disease and congestive heart failure, patients undergoing transcatheter aortic valve replacement, and patients undergoing PCI including in-hospital and long-term adverse events (3, 5, 8, 10–12). In the context of acute coronary syndrome, anemia exacerbates the mismatch between myocardial oxygen supply and demand by reducing oxygen-carrying capacity and simultaneously increasing myocardial consumption through increased cardiac output (13).

In recent years, there has been growing interest in PCI in anatomically complex lesions and higher-risk patients, particularly those with comorbidities and compromised hemodynamic status such as low ejection fraction (14). Impella (Abiomed Inc., Danvers, MA, USA), which is a percutaneous left ventricular assist device (pLVAD), has been increasingly used for mechanical circulatory support during PCI to provide hemodynamic support and enable complete revascularization (15, 16).

Large-bore access procedures such as Impella-supported high-risk PCI (HRPCI) can increase the risk of bleeding. Despite its prevalence and prognostic impact, there are currently no clear guidelines for the management of anemia in PCI patients. As such, anemia has been overlooked in the context of HRPCI. Recognizing this gap, we aimed to investigate the characteristics of anemia patients and assess the association between anemia and adverse outcomes in patients undergoing HRPCI using data from the cVAD PROTECT III study.

## Methods

### Study design, population, and oversight

**Figure 1:**
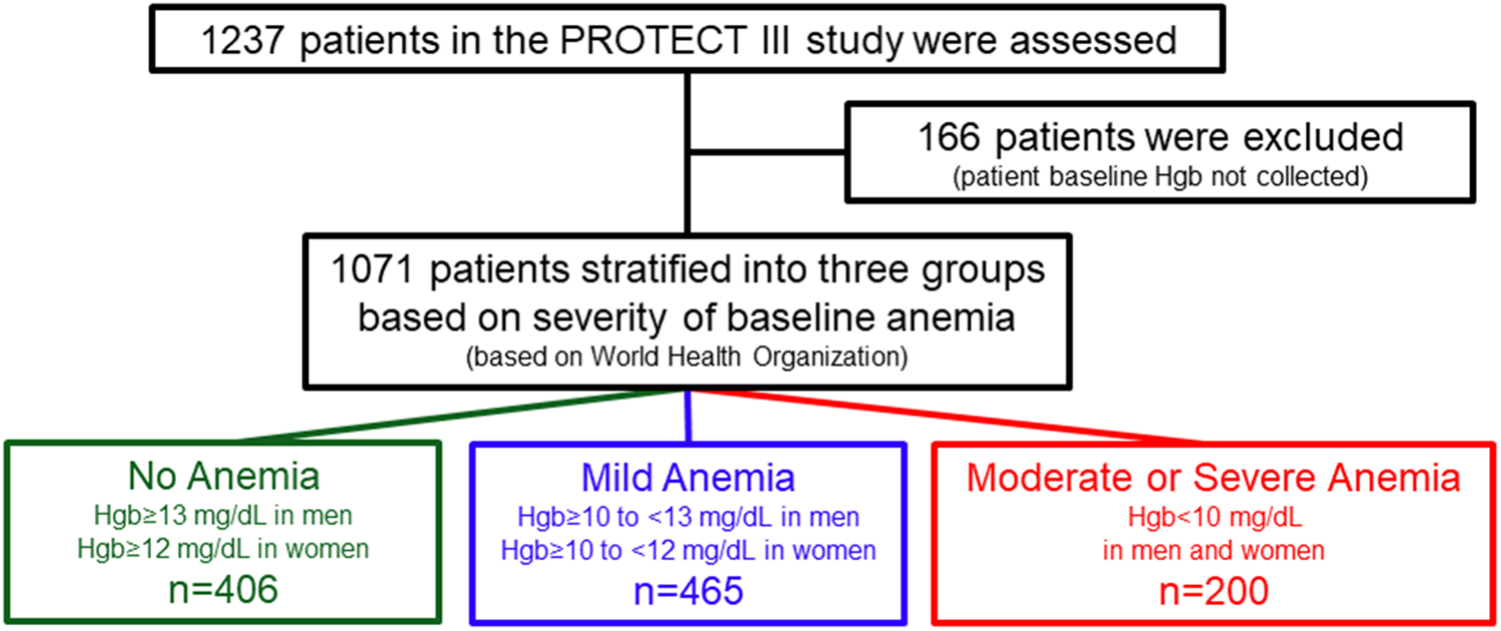
Study flowchart. World Health Organization (2000) has defined mild anemia as hemoglobin 11–11.9 g/dL in women and 11–12.9 g/dL in men; moderate anemia as hemoglobin 8–10.9 g/dL; and severe anemia as hemoglobin <8 g/dL.

The PROTECT III study design, rationale, and preliminary results have been previously published (17). In brief, the PROTECT III study is a single-arm, observational study that enrolled 1,237 patients between March 2017 and March 2020. These patients underwent Impella-supported HRPCI across 46 centers in North America. This study is part of the global cVAD registry (NCT04136392), including several separate post-approval studies intended to evaluate the safety and efficacy of Impella mechanical circulatory support use for different cardiovascular conditions (18). The PROTECT III study is an FDA-audited post-approval study on patients undergoing elective or urgent HRPCI procedures (i.e., without cardiogenic shock) and in whom Impella 2.5 or Impella CP was implanted for hemodynamic support during the procedure. The decision to use Impella and the definition of HRPCI were made according to the physician's standard of care. Patients met enrollment criteria once the decision was made to use Impella before or during the index PCI. Bailout pLVAD implantation cases were excluded. The current analysis includes only patients with baseline hemoglobin available. The derivation of the analysis population is described in Figure 1.

The study was conducted in accordance with the Declaration of Helsinki and was approved by the applicable Institutional Review Board or Independent Ethics Committee at each participating site prior to enrollment. Baseline demographics, blood tests, and echocardiography were collected before the index procedure and patients were followed for 90 days. Angiographic data analysis was performed by an independent core lab (Beth Israel Deaconess Medical Center Angiographic Core Laboratory). An independent 12-member steering committee oversaw the conduct of the cVAD study. An independent Clinical Events Committee adjudicated major adverse cardiovascular and cerebrovascular events (MACCE: composite of all-cause mortality, myocardial infarction, stroke/transient ischemic attack (TIA), and repeat revascularization), in addition to adjudicating the relatedness to the study procedure and/or device. The sponsor (Abiomed Inc., Danvers, MA, USA) oversaw study data management and source document verification and provided funding to the Cardiovascular Research Foundation (New York, NY, USA) for statistical analysis. The authors had unrestricted access to the study data and accepted responsibility for the integrity of this report. Artificial intelligence was not utilized at any point throughout the generation of this manuscript.

### Definitions

Anemia was defined according to World Health Organization (WHO) criteria (9). The patients were categorized into three groups: no anemia, characterized by Hgb levels ≥12 mg/dL for women and ≥13 mg/dL for men; mild anemia, with Hgb levels ≥10 to <12 mg/dL in women and ≥10 to <13 mg/dL in men; and moderate or severe anemia, indicated by Hgb levels <10 mg/dL in men and women.

The primary endpoint was MACCE at 30 and 90 days. Secondary endpoints included the following: (1) major bleeding events [Bleeding Academic Research Consortium (BARC) ≥ 3a], (2) duration of hospitalization, (3) major vascular or structural complications requiring surgery or intervention, and (4) 1-year all-cause mortality. Detailed definitions of endpoints have been previously published (19). Prolonged Impella support was defined by any of the following conditions: the site reported that the Impella device was not removed at the end of the procedure, the duration from Impella initiation to PCI completion exceeded 60 min, or additional mechanical support devices were implanted after the Impella was removed.

### Statistical analysis

The baseline characteristics were summarized as mean ± standard deviation or median and interquartile range (IQR) for continuous measures and proportions for categorical variables. Between the study groups, categorical variables were summarized as percentages and compared using the chi-squared or Fisher's exact test (e.g., blood transfusion, bleeding, and vascular complications requiring intervention), whereas continuous variables (e.g., duration of hospitalization) were summarized as mean ± standard deviation and median (IQR) and compared using ANOVA and Wilcoxon rank-sum test. For time-to-first event analyses, event rates were estimated by the Kaplan–Meier method and compared using the log-rank test. We tested the normality of continuous variables using the Shapiro–Wilk test. If normality failed per the Shapiro–Wilk test (*p* < 0.05), then the distributions were compared using the Wilcoxon rank-sum test. The Cox proportional hazard model is applied to compare anemia groups and their association with 30-day and 90-day MACCE rates after adjustment for sex, age, left ventricular ejection fraction (LVEF), and eGFR.

Multivariable logistic regression was done to compare anemia groups and bleeding events after adjustment for the same confounders as in the Cox regression model. Additional logistic regression analysis was performed to compare the need for blood transfusion after adjustment for age, sex, anticoagulation treatment, dialysis, peripheral artery disease, and prolonged Impella support between anemia groups. The variables included in the multivariable Cox proportional hazard regression for 30-day and 90-day MACCE, and those included in multivariable logistic regression models were selected based on prior evidence in the literature and their effect in univariable analysis.

An additional sensitivity analysis excluding patients on dialysis was performed for the primary endpoint and 1-year mortality using Kaplan–Meier time-to-event estimation. To compare between anemia groups MACCE rates at 30- and 90-day Cox proportional hazards regression model excluding patients on dialysis was completed and adjusted to the same confounders as in the entire study population. Multiple imputation was used to account for missing data for covariates in the multivariable Cox and logistic regression analyses. For this analysis, we used the Markov chain Monte Carlo method. All *p*-values are two-tailed, and *p* < 0.05 was considered significant for all analyses. Statistical analyses were performed using SAS version 9.4 (SAS Institute Inc., Cary, NC).

### Data transparency and openness

Because of the sensitive nature of the data collected for this study, requests to access the dataset from qualified researchers trained in human subject confidentiality protocols may be sent to the study sponsor (Abiomed) at aalmedhychy@abiomed.com.

## Results

### Baseline characteristics

From March 2017 to March 2020, 1,237 consecutive patients were enrolled in the PROTECT III study at 46 sites in North America, of whom 1,071 had baseline Hgb available (Figure 1). At baseline, 37.9% of patients (406) had no anemia, 43.4% (465) had mild anemia, and 18.7% (200) had moderate to severe anemia. Baseline patient characteristics are presented in Table 1. Patients with anemia, compared to those without, were characterized by older age and a higher burden of comorbidities including chronic kidney disease, peripheral vascular diseases, and diabetes mellitus. In addition, the proportion of female patients was higher in the two anemia groups than in the no anemia group, although males were predominant in all groups. There were no significant differences in the rates of prior PCI or previous coronary artery bypass grafting between the three groups. Patients with anemia were more likely to have lower left ventricular ejection fraction (LVEF) (ANOVA *p*-value = 0.002), but there were no significant differences in right ventricle function or valvular pathologies.

**Table 1:** Baseline demographics and procedural characteristics according to anemia severity.

	No anemia	Mild anemia	Moderate to severe anemia	Overall *p*-value
	(*n* = 406)	(*n* = 465)	(*n* = 200)	
Demographics				
Age, year	69.2 ± 11.7	72.2 ± 10.9	71.5 ± 10.3	0.0003
Sex, male	76.4 (310/406)	73.1 (340/465)	66.0 (132/200)	0.03
Race				
White or Caucasian	71.7 (291/406)	64.3 (299/465)	63.0 (126/200)	0.03
Black or African American	8.1 (33/406)	15.5 (72/465)	18.0 (36/200)	0.0005
Asian	3.4 (14/406)	3.2 (15/465)	3.5 (7/200)	0.98
American Indian or Alaska Native	0 (0/406)	1.1 (5/465)	0 (0/200)	0.04
Native Hawaiian/other Pacific Islander	0 (0/406)	0 (0/465)	0.5 (1/200)	0.11
Other race	4.4 (18/406)	3.0 (14/465)	3.5 (7/200)	0.53
Body mass index, kg/m^2^	28.9 ± 6.2	28.5 ± 6.6	28.5 ± 7.0	0.55
Medical history				
History of tobacco use	59.1 (234/396)	66.6 (303/455)	58.6 (112/191)	0.04
Hypertension	89.1 (360/404)	92.6 (427/461)	95.5 (190/199)	0.02
Dyslipidemia	76.8 (307/400)	82.8 (380/459)	77.2 (152/197)	0.06
Diabetes	48.5 (195/402)	59.7 (276/462)	66.3 (132/199)	<0.0001
Peripheral vascular disease	15.8 (63/400)	23.2 (106/457)	32.7 (64/196)	<0.0001
Chronic pulmonary disease	17.2 (69/401)	26.2 (120/458)	24.5 (47/192)	0.005
Stroke/TIA	14.3 (57/400)	19.3 (89/460)	18.4 (36/196)	0.13
Renal insufficiency	18.3 (73/399)	35.6 (164/461)	53.3 (105/197)	<0.0001
eGFR^a^, mL/min/1.73 m^2^	73.9 ± 22.4	65.7 ± 23.5	59.2 ± 27.8	<0.0001
On dialysis	4.1 (3/73)	32.9 (54/164)	46.7 (49/105)	<0.0001
Prior myocardial infarction	37.7 (147/390)	41.1 (183/445)	43.5 (81/186)	0.36
Prior coronary artery bypass grafting	16.1 (65/403)	14.3 (66/463)	9.7 (19/196)	0.10
Heart failure	56.5 (225/398)	60.2 (277/460)	60.4 (119/197)	0.49
Left ventricular ejection fraction, %	32.2 ± 15.7	34.9 ± 15.5	37.2 ± 14.6	0.002
Atrial fibrillation	41.9 (13/31)	36.8 (14/38)	15.8 (3/19)	0.15
Indication for PCI				
Acute coronary syndrome	55.0 (198/360)	60.8 (245/403)	68.0 (123/181)	0.01
Urgent PCI	51.4 (209/407)	44.1 (260/465)	40.5 (81/200)	0.72
Number of diseased vessels				
1	12.2 (49/402)	11.1 (51/460)	8.1 (16/198)	0.31
2	31.3 (126/402)	31.7 (146/460)	26.8 (53/198)	0.42
3	56.0 (225/402)	55.4 (255/460)	62.1 (123/198)	0.25
Left main disease	51.6 (208/403)	62.3 (288/462)	64.0 (126/197)	0.001
Number of vessels treated				0.77
1	27.7 (106/383)	29.5 (128/434)	25.8 (47/182)	0.63
2	46.2 (178/383)	44.5 (193/434)	44.0 (80/182)	0.84
3	26.1 (100/383)	26.0 (113/434)	30.2 (55/182)	0.52
Pre-PCI SYNTAX score	26.9 ± 12.3	28.2 ± 13.1	30.3 ± 11.8	0.04
Post-PCI SYNTAX score	6.5 ± 8.0	7.1 ± 9.2	6.1 ± 7.0	0.50
Pre-PCI ischemia jeopardy score	8.7 ± 2.2	8.9 ± 2.1	9.1 ± 2.2	0.09
Post-PCI ischemia jeopardy score	1.8 ± 1.9	2.0 ± 2.2	1.9 ± 2.2	0.48

Data are presented as mean ± standard deviation (*n*) or % (*n*/*N*), where applicable.

eGFR, estimated glomerular filtration rate; PCI, percutaneous coronary intervention; SYNTAX, synergy between percutaneous coronary intervention with taxus and cardiac surgery; TIA, transient ischemic attack.

^a^ eGFR was calculated using the 2021 CKD-EPI creatinine equation.

### Admission and procedural characteristics

Patients with moderate to severe anemia had higher rates of acute coronary syndrome as the primary reason for admission compared to patients with mild or no anemia (68.0% vs. 60.8% vs. 55.0% respectively, chi-squared *p*-value = 0.01).

Both patients with moderate to severe anemia and those with mild anemia had a significantly higher incidence of left main (LM) disease compared to patients with no anemia (64.0% vs. 62.3% vs. 51.6% respectively, chi-squared *p*-value = 0.001). Patients with baseline anemia also had higher pre-PCI SYNTAX scores, longer duration of PCI procedure, and longer hospitalization time with greater need for blood transfusion compared to patients with no anemia (Table 2). However, there was no significant difference in post-PCI SYNTAX score or Impella support duration between groups.

**Table 2:** Immediate PCI-related complications and in-hospital adverse events according to anemia severity.

	No anemia (*n* = 406)	Mild anemia (*n* = 296)	Moderate to severe anemia (*n* = 159)	Overall*p*-value
Duration of index PCI procedure (hours)	1.9 ± 1.0	2.1 ± 1.1	2.2 ± 1.2	0.001
Duration of hospitalization (days)	5.0 [2.0, 9.0]	7.0 [3.0, 11.0]	10.0 [5.0, 16.0]	<0.0001
PCI-related complications^a^	4.6 (17/369)	4.7 (20/424)	2.2 (4/179)	0.34
No reflow	11.8% (2/17)	5% (1/20)	0% (0/4)	0.62
Abrupt closure	5.9% (1/17)	5% (1/20)	0% (0/4)	0.89
Dissection	11.8% (2/17)	5% (1/20)	0% (0/4)	0.62
Distal embolization	0% (0/17)	5% (1/20)	0% (0/4)	0.58
Perforation	23.5% (4/17)	40% (8/20)	75% (3/4)	0.14
Adverse events				
Pericardial effusion requiring pericardiocentesis	2.0 (4/406)	0.9 (4/465)	2.0 (4/199)	0.41
Cardiac arrest	1.2 (5/406)	1.9 (9/465)	4.5 (9/199)	0.03
Cardiogenic shock	2.0 (8/406)	2.4 (11/465)	1.0 (2/199)	0.51
Ventricular arrhythmia	1.5 (6/406)	1.1 (5/465)	2.0 (4/199)	0.63
AKI (Stages 2 or 3)	4.2 (17/407)	4.3 (20/465)	5.0 (10/199)	0.89
AKI (Stages 2 or 3) excluding dialysis patients^b^	4.2 (17/403)	4.9 (20/411)	6.7 (10/150)	0.49
Life-threatening/disabling/major bleeding (BARC ≥3a)	1.2 (5/406)	2.4 (11/465)	5.0 (10/199)	0.02
Life-threatening/disabling/major bleeding (BARC ≥3a) excluding dialysis patients^b^	1.0 (4/403)	2.2 (9/411)	6.0 (9/150)	0.002
Hemolysis	1.5 (6/406)	1.5 (7/465)	0 (0/199)	0.22
Thrombocytopenia	0.7 (3/406)	0.2 (1/465)	3.5 (7/199)	0.0004
Anemia requiring transfusion	2.2 (9/406)	8.0 (37/465)	19.6 (39/199)	<0.0001
Anemia requiring transfusion excluding dialysis patients^b^	2.2 (9/403)	8.3 (34/411)	20.7 (31/150)	<0.0001
Vascular/cardiac structural complication requiring surgery/reintervention	1.5 (6/406)	0.4 (2/465)	1.5 (3/199)	0.24
Access site-related hematoma	7.1 (29/406)	7.5 (35/465)	8.0 (16/199)	0.92

Data are presented as % (*n*/*N*) or median (IQR), where applicable.

AKI, acute kidney injury; BARC, bleeding academic research consortium; PCI, percutaneous coronary intervention.

^a^ PCI-related complications are defined as the composite of no reflow, abrupt closure, dissection, distal embolus, and perforation during PCI.

^b^ Sensitivity analysis was conducted excluding dialysis patients and using the same statistical methods as explained in methods for all the cohorts.

### Major adverse cardiovascular and cerebrovascular events

**Figure 2:**
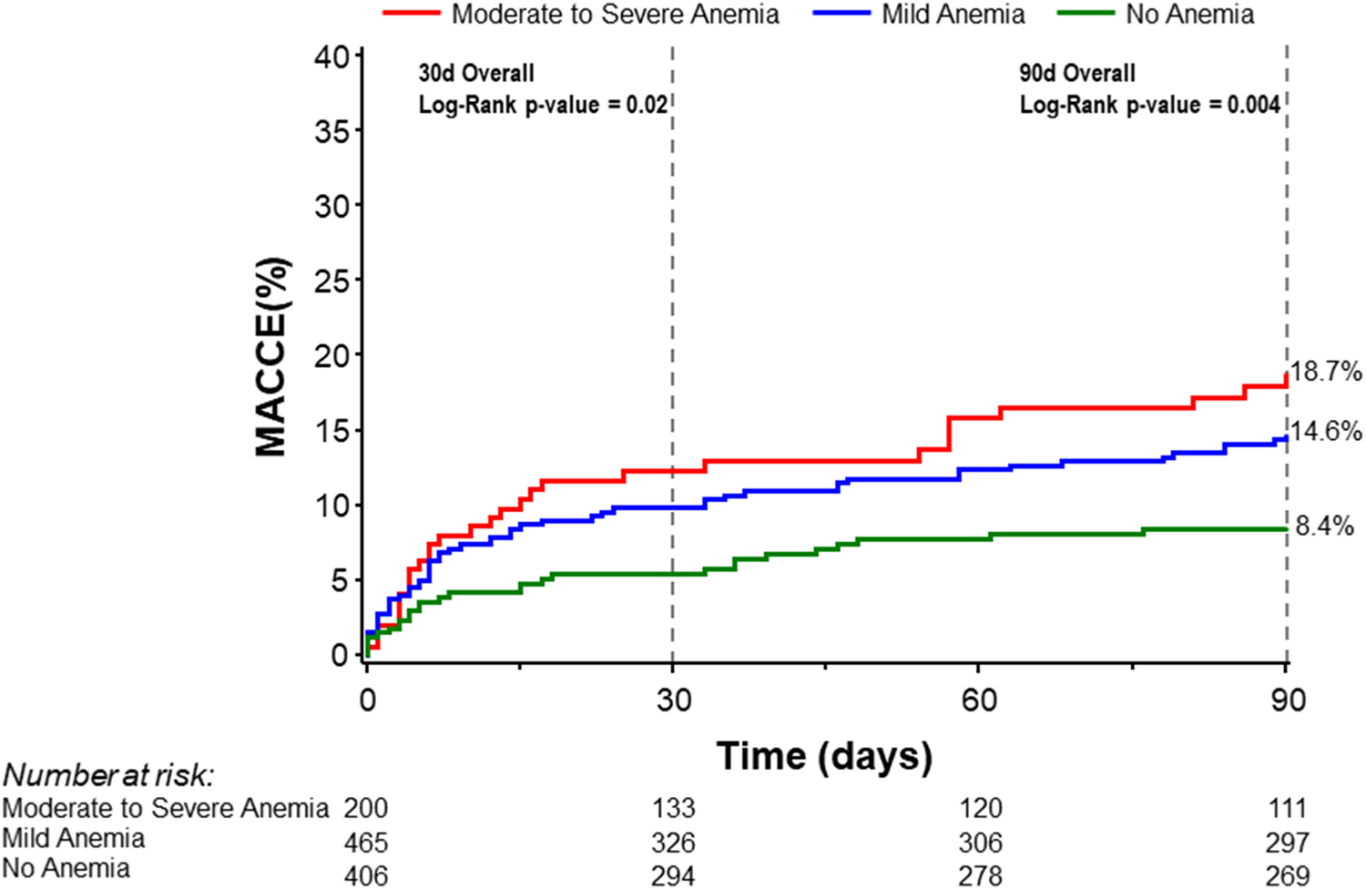
Kaplan–Meier curves for 30- and 90-day MACCE stratified by severity of anemia. MACCE denotes major adverse cardiovascular and cerebrovascular events.

**Figure 3:**
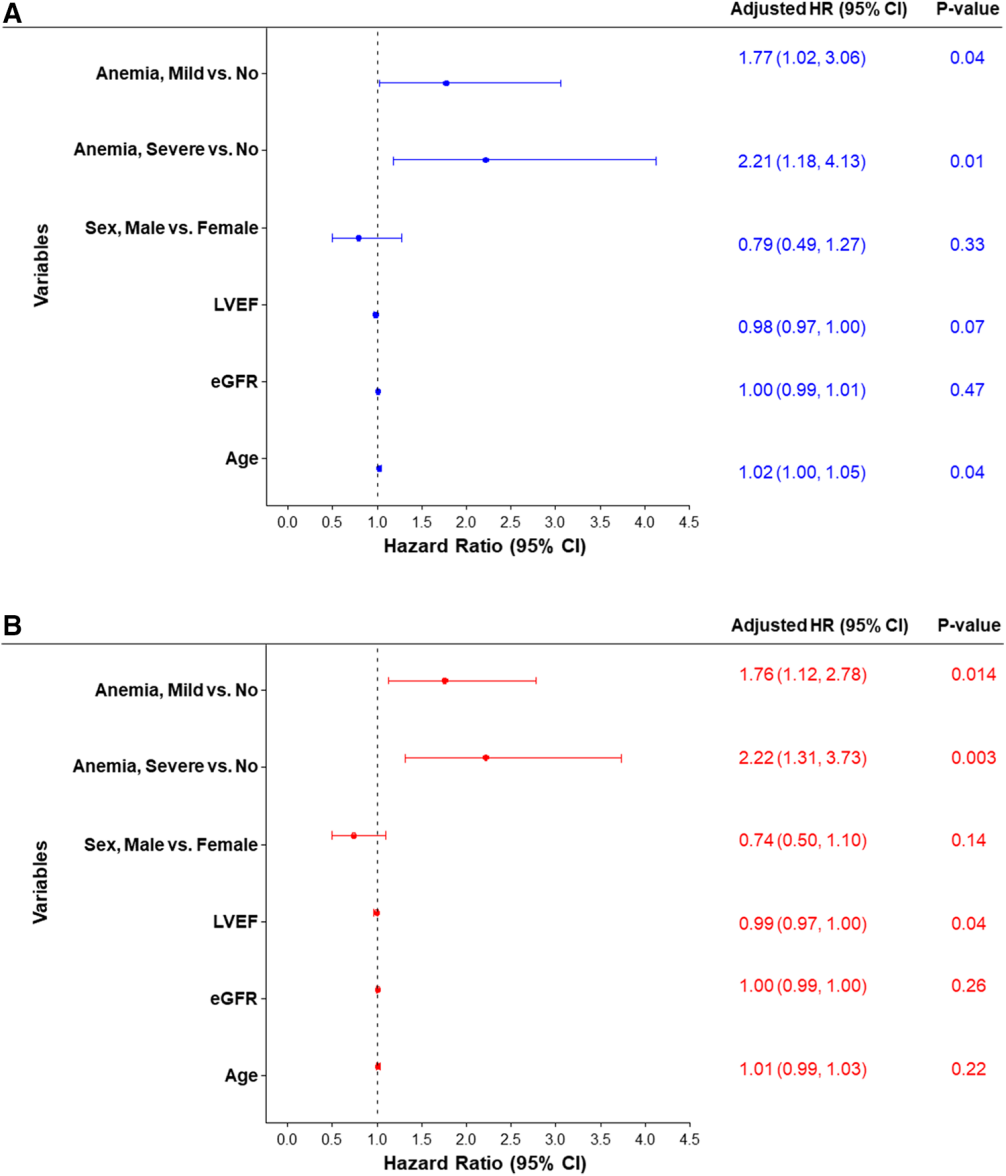
Forest plot of the adjusted hazard ratio for MACCE rates estimated by cox proportional hazards regression models at (**A**) 30 days and (**B**) 90 days. eGFR, estimated glomerular filtration rate; LVEF, left ventricular ejection fraction.

Univariate analysis was performed for 30-day and 90-day MACCE. At 30 days, patients with baseline moderate to severe anemia had significantly higher MACCE rates compared to patients with mild anemia or no anemia (12.3% vs. 9.8% vs. 5.4% respectively, overall log-rank *p*-value = 0.02). This difference persisted at 90 days with MACCE rates among the moderate to severe anemia group being 18.7% compared to 14.6% in patients with mild anemia and 8.4% in patients with no anemia (overall log-rank *p*-value = 0.004; Figure 2, Table 3). Multivariable analysis was then performed adjusting for age, sex, LVEF, and eGFR. The findings from multivariable analysis remained consistent showing that both mild and moderate to severe anemia were associated with higher MACCE rates at 30 and 90 days (Figure 3). A sensitivity analysis excluding patients with dialysis was conducted. Of the 1,071 patients with baseline hemoglobin measurements, 106 patients were reported to be on dialysis. Among them, 46.7% (49 patients) had moderate to severe anemia. In this analysis unadjusted MACCE rates remained higher among patients with anemia at 30 days (log-rank *p* = 0.046) and 90 days (log-rank *p* = 0.02) (Supplementary Table S1 and Figure S1), as well as all-cause mortality at 1 year (log-rank *p* = 0.002) (Supplementary Table S1 and Figure S2). However, after adjusting for confounders (age, sex, left ventricular ejection fraction, eGFR, and anemia group) MACCE rates are significantly higher among the moderate to severe anemia group at 90 days and non-significant at 30 days, moreover mild anemia was not a significant predictor of MACCE outcomes in this adjusted model (Supplementary Figure S3).

**Table 3:** Ninety-day major adverse cardiac and cerebrovascular events at 90 days and mortality at 1 year according to anemia severity.

	No anemia	Mild anemia	Moderate to severe anemia	Overall *p*-value
30-day MACCE^a^	5.4% (19)	9.8% (40)	12.3% (22)	0.02
Death	4.3% (14)	8.5% (34)	10.2% (18)	0.02
Non-cardiovascular	0.3% (1)	1.1% (4)	1.4% (2)	0.42
Cardiovascular	4.0% (13)	7.5% (30)	8.9% (16)	0.04
Myocardial infarction	1.3% (5)	2.1% (8)	2.3% (4)	0.76
Stroke/TIA	1.4% (5)	1.8% (8)	0.5% (1)	0.43
Repeat revascularization	0.3% (1)	0.8% (3)	1.9% (3)	0.21
90-day MACCE^a^	8.4% (28)	14.6% (57)	18.7% (31)	0.004
Death	7.2% (23)	11.4% (44)	16.5 (27)	0.007
Non-cardiovascular	0.3% (1)	1.4% (5)	2.9% (4)	0.09
Cardiovascular	6.9% (22)	10.1% (39)	14.0% (23)	0.03
Myocardial infarction	2.1% (7)	3.9% (14)	4.6% (7)	0.33
Stroke/TIA	1.4% (5)	2.1% (9)	0.5% (1)	0.32
Repeat revascularization	1.0% (3)	3.0% (10)	3.5% (5)	0.14
1-year mortality	15.5% (46)	23.2% (84)	31.8% (47)	0.0002

Values are Kaplan–Meier event rates (*n* of events). Event rates are Kaplan–Meier event rates and are compared by the log-rank test.

MACCE, major adverse cardiovascular and cerebrovascular events; TIA, transient ischemic attack.

^a^ MACCE is defined as the composite of all-cause death, myocardial infarction, stroke/TIA, and revascularization.

### Secondary endpoints

**Figure 4:**
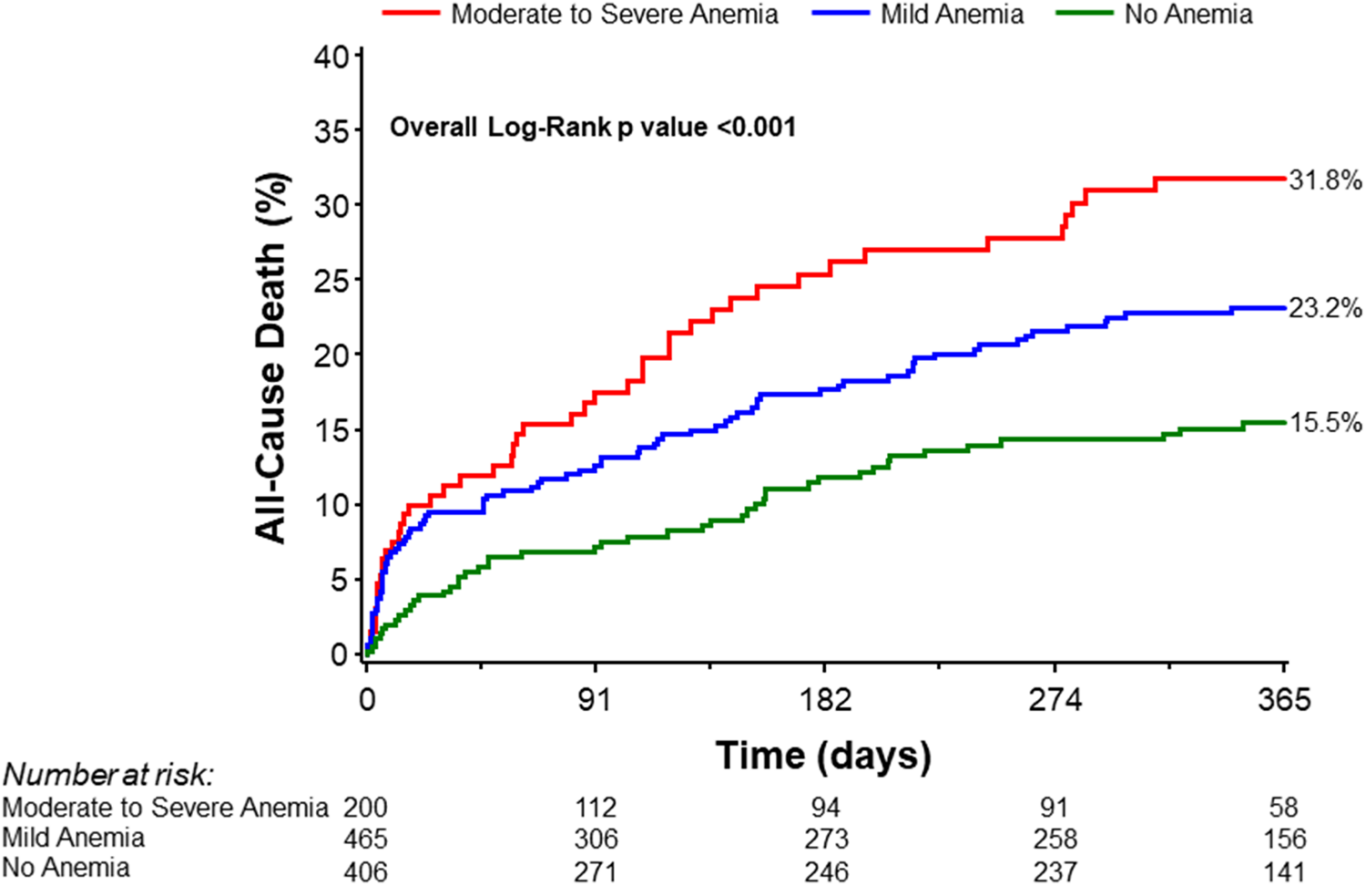
Kaplan–Meier curves for all-cause mortality at 1 year stratified by severity of anemia.

A greater proportion of patients with moderate to severe anemia and mild anemia had major bleeding incidents (BARC ≥ 3a) compared to patients with no anemia (5.0% vs. 2.4% vs. 1.2%, Chi-square *p*-value = 0.02; Table 2). Moreover, blood transfusion was significantly higher among anemic patients (Chi-square *p*-value <0.0001). This difference remained statistically significant after excluding patients on dialysis (Chi-square *p*-value <0.0001; Table 2). Patients with moderate to severe anemia were hospitalized longer compared to mild anemia or no anemia groups [10.0 (5.0, 16.0) days vs. 7.0 (3.0, 11.0) days vs. 5.0 (2.0, 9.0), Wilcoxon rank-sum *p*-value <0.0001; Table 2]. There were no significant differences in major vascular complications requiring intervention. Notably, one-year mortality was significantly higher in the moderate to severe anemia group than in the mild and no anemia group (31.8%, 23.2%, and 15.5%, respectively; log-rank *p*-value = 0.0002; Table 3, Figure 4).

**Figure 5:**
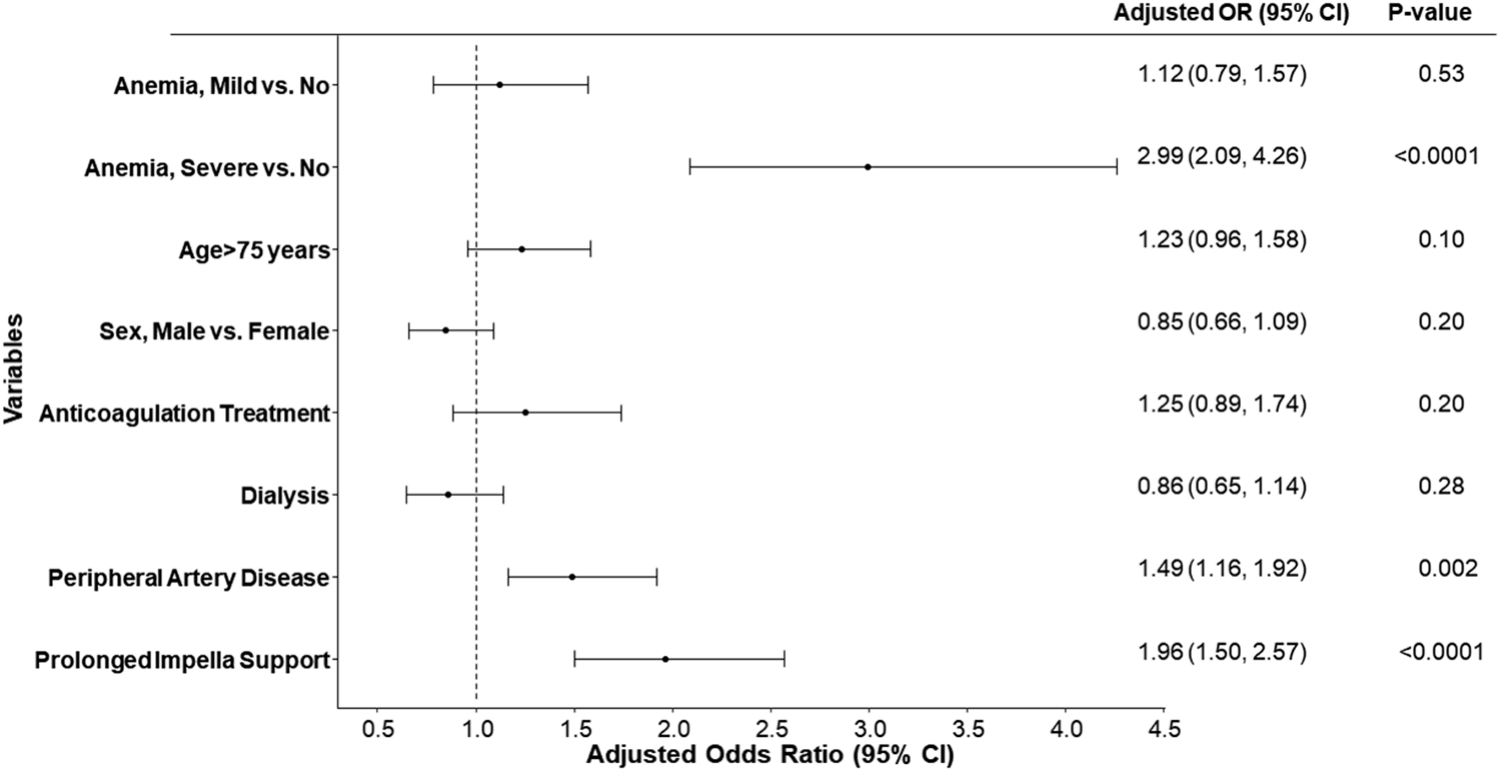
Forest plot of the adjusted odds ratio for blood transfusion for different variables. Odds ratios (OR) and 95% confidence intervals (95% CI) are estimated by multiple logistic regression models. Prolonged support Impella is defined as any Impella support beyond the index procedure.

Multiple logistic regression models adjusting for age, sex, LVEF, eGFR, and anemia group indicated that moderate to severe anemia was independently associated with major bleeding [Adjusted OR (95% CI): 2.53 (1.37, 4.68), *p*-value = 0.003] (Supplementary Table S2). Further analysis on predictors of blood transfusion after adjusting for multiple covariates (age, anticoagulation treatment, dialysis, peripheral artery disease, sex, prolonged Impella support, and anemia group) revealed that moderate or severe anemia, peripheral vascular disease, and prolonged Impella support were independently associated with blood transfusion requirements (Figure 5).

## Discussion

In this study assessing outcomes in pLVAD-supported HRPCI, we found that baseline anemia is common and associated with MACCE, bleeding events, and death in this population.

Several previous studies have indicated an association between baseline anemia and adverse outcomes in contemporary PCI with a twofold increased risk of mortality and MACE events, as well as an elevated risk of bleeding with an incremental decrease in hemoglobin level (20). Similarly, a report from the Myocardial Infarction Data Acquisition System suggests a higher 1-year mortality among patients with vs. without anemia (21). However, other studies found no increase in risk after adjustment for age, comorbidity burden, and procedural demographics (22, 23). The association between baseline anemia and outcomes in Impella-supported HRPCI has not previously been examined. Based on our results, anemia should be considered in the risk stratification of patients intended for HRPCI.

There is an ongoing debate about whether anemia is simply a marker of sicker more medically complex patients or an independent predictor of adverse clinical outcomes. Within our cohort, anemia was more prevalent in patients with underlying comorbidities. However, this increased risk persisted in the moderate to severe anemia group after adjustment for potential confounding factors (age, renal function, LVEF, and sex). Given the high prevalence of anemia among dialysis patients (24) and that dialysis is independently associated with increased risk for adverse cardiovascular events and procedure complications (25–27), we conducted a sensitivity analysis excluding dialysis patients. The result of this analysis showed that only moderate to severe anemia was independently associated with higher MACCE rates at 90 days. This suggests that, for this patient group, lower hemoglobin cutoffs should be considered in risk assessments compared to non-dialysis patients. These findings suggest that this association between anemia and MACCE rates might be affected by other confounders and is more evident in patients with moderate or severe anemia rather than mild anemia.

Several hypotheses may account for the observed association between anemia and adverse PCI outcomes. An imbalance between myocardial oxygen supply and demand may be exacerbated by anemia due to the combination of reduced oxygen-carrying capacity and increased myocardial oxygen consumption through increased cardiac output associated with severe anemia and obstructive coronary disease. Additionally, there is a link between nitric oxide and Hgb, further influencing vascular function and causing impaired vascular relaxation and healing, which is exacerbated by an inflammatory response that is inversely correlated with anemia in acute coronary syndrome patients (13).

We also investigated the impact of anemia on major bleeding events (BARC ≥ 3a) and blood transfusions. Results of a logistic regression model for blood transfusion showed that moderate to severe anemia prolonged Impella support and peripheral vascular disease were independent factors associated with blood transfusion. This suggests that patients with baseline anemia have more hemolysis during Impella support and impaired hemostasis worsening their existing baseline anemia and necessitating blood transfusion. These findings raise the question of whether pre-procedure blood transfusion should be considered in certain cases along with a more meticulous access and closure technique planning to minimize procedural complications. Periprocedural anticoagulation strategy and choice of antiplatelets should be carefully assessed weighing the risks vs. benefits. Additionally, given that prolonged Impella support was also associated with blood transfusion, it may suggest that removal of Impella should be done as promptly as possible once the patient is stabilized and no longer in need of hemodynamic support.

Current clinical guidelines lack recommendations for the concurrent management of anemia in patients undergoing PCI or large-bore procedures such as Impella-supported HRPCI, other than advising measures to minimize bleeding risks and utilizing risk scores to guide dual antiplatelet therapy. Regarding blood transfusion treatment in general, the recent randomized MINT study, comparing restrictive or liberal transfusion strategies in myocardial infarction and anemia, found that the liberal transfusion strategy did not significantly reduce the risk of recurrent myocardial infarction or death at 30 days (28). It should be noted that this trial also included patients who did not have PCI. The lack of specific guidelines and integration of formal bleeding risk algorithms into routine care renders the management of patients with anemia who require high-risk PCI difficult.

### Study limitations

Our study has several limitations. First, this is a *post hoc* analysis derived from a single-arm observational study and thus causality cannot be determined. Additionally, the management and treatment of anemia were at the discretion of the operators and therefore may have varied both between individual operators and across medical centers.

In conclusion, our study shows that moderate to severe anemia is an independent predictor of MACCE and bleeding after Impella-supported complex PCI, portending a worse prognosis. The extent to which these associations between anemia and adverse events after complex PCI can be mitigated by optimized anemia management and tailored interventions remains to be established.

## Data Availability

The datasets presented in this article are not readily available because of the sensitive nature of the data collected for this study. Requests to access the datasets from qualified researchers trained in human subject confidentiality protocols should be directed to the study Sponsor (Abiomed) at aalmedhychy@abiomed.com.
