# Supplementary material for: Implications of anemia in patients undergoing PCI with Impella-support: insights from the PROTECT III study
Source: Front Cardiovasc Med. 2024 Jul 18;11:1429900. doi: 10.3389/fcvm.2024.1429900 (PMC11291217; doi:10.3389/fcvm.2024.1429900)
Supplement: Supplementary file 1 [file Datasheet1.docx]

**Implications of Anemia on Patients Undergoing PCI with Impella-Support**

**Insights From the PROTECT III Study**

Batla Falah, MD; Björn Redfors, MD, PhD; Duzhi Zhao, MS; Aditya S. Bharadwaj, MD; Mir B. Basir, DO; Julia B. Thompson, MS; Rajan A. Patel, MD; Michael J. Schonning, MS, MBS; Arsalan Abu-Much, MD; Yiran Zhang, MS; Wayne B. Batchelor, MD, MHS; Cindy L. Grines, MD; William W. O’Neill, MD

**Supplemental Appendix**

| **Title** | **Page Number** |
| --- | --- |
| **Supplemental Table S1.** Major Adverse Cardiac and Cerebrovascular Events at 90 days and Mortality at 1 year According to Anemia Severity excluding patients on dialysis. | 2 |
| **Supplemental Table S2.** Risk Factors Associated with Life Threatening Bleeding | 3 |
| **Supplemental Figure S1.** Kaplan Meier Curves for 30 and 90 days MACCE stratified by severity of anemia excluding patients on dialysis. | 4 |
| **Supplemental Figure S2.** Kaplan Meier Curves for all-cause mortality at 1 year stratified by severity of anemia excluding patients on dialysis**.** | 5 |
| **Supplemental Figure S3.** Forest Plot of the Adjusted Hazards Ratio for MACCE Rates at 30 and 90 Days Estimated by Cox Proportional Hazards Regression Model excluding patients on dialysis. | 6 - 7 |

**Supplemental Table S1. Major Adverse Cardiac and Cerebrovascular Events at 90 days and Mortality at 1 year According to Anemia Severity excluding patients on dialysis.**

|  | No Anemia | Mild Anemia | Moderate to Severe Anemia | Overall  P value |
| --- | --- | --- | --- | --- |
| *30-day MACCE** | 5.5% (19) | 9.5% (34) | 12.0% (16) | 0.046 |
| Death | 4.3% (14) | 8.5% (30) | 8.9% (12) | 0.04 |
| Non-cardiovascular | 0.3% (1) | 1.2% (4) | 0.9% (1) | 0.41 |
| Cardiovascular | 4.0% (13) | 7.4% (26) | 8.2% (11) | 0.08 |
| Myocardial Infarction | 1.4% (5) | 2.0% (7) | 1.6% (2) | 0.85 |
| Stroke/TIA | 1.4% (5) | 1.3% (5) | 0.7% (1) | 0.82 |
| Repeat Revascularization | 0.3% (1) | 0.9% (3) | 1.8% (2) | 0.35 |
| *90-day MACCE** | 8.4% (28) | 14.0% (48) | 17.4% (22) | 0.02 |
| Death | 7.3% (23) | 10.8% (37) | 15.3% (19) | 0.03 |
| Non-cardiovascular | 0.3% (1) | 1.2% (4) | 2.8% (3) | 0.12 |
| Cardiovascular | 7.0% (22) | 9.7% (33) | 12.8% (16) | 0.12 |
| Myocardial Infarction | 2.1% (7) | 3.8% (12) | 4.5% (5) | 0.43 |
| Stroke/TIA | 1.4% (5) | 1.6% (6) | 0.7% (1) | 0.74 |
| Repeat Revascularization | 1.1% (3) | 3.4% (10) | 2.8% (3) | 0.15 |
| *1-Year Mortality* | 15.7% (46) | 20.9% (67) | 30.3% (34) | 0.0002 |

Values are Kaplan-Meier event rates (n of events). Event rates are Kaplan-Meier event rates and compared by the log-rank test. *MACCE is defined as the composite of all-cause death, myocardial infarction, stroke/TIA, and revascularization. MACCE denotes major adverse cardiovascular and cerebrovascular events; TIA, transient ischemic attack.

**Supplemental Table S2. Risk Factors Associated with Life Threatening or Major Bleeding at Discharge.**

| **Variable** | **Adjusted OR (95% CI)** | **p-value** |
| --- | --- | --- |
| Age >75 years | 1.04 (0.99, 1.09) | 0.12 |
| eGFR^*^ | 1.00 (0.98, 1.02) | 0.87 |
| LVEF | 1.00 (0.97, 1.03) | 0.89 |
| Sex, Male vs. Female | 0.98 (0.61, 1.57) | 0.92 |
| Anemia, Severe vs. No | 2.53 (1.37, 4.69) | 0.003 |
| Anemia, Mild vs. No | 0.88 (0.49, 1.60) | 0.68 |

Odds ratios (OR) and 95% confidence intervals (95% CI) are estimated by Multiple logistic regression models. Multiple imputation using Markov Chain Monte Carlo (MCMC) method was applied to all variables in the model except for anemia status. ^*^eGFR was calculated using 2021 CKD-EPI Creatinine Equation.

**Supplemental Figure S1.** **Kaplan Meier Curves for 30 and 90 days MACCE stratified by severity of anemia excluding patients on dialysis.**


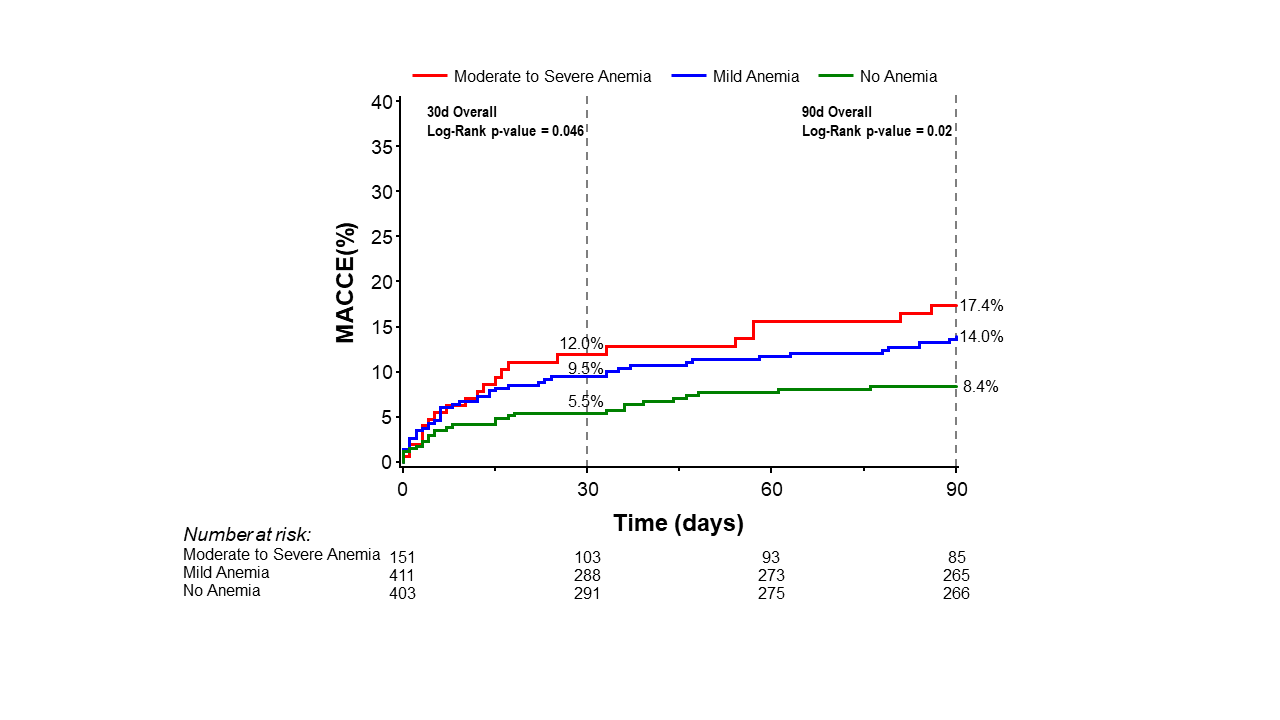


**Supplemental Figure S2. Kaplan Meier Curves for all-cause mortality at 1 year stratified by severity of anemia excluding patients on dialysis.**


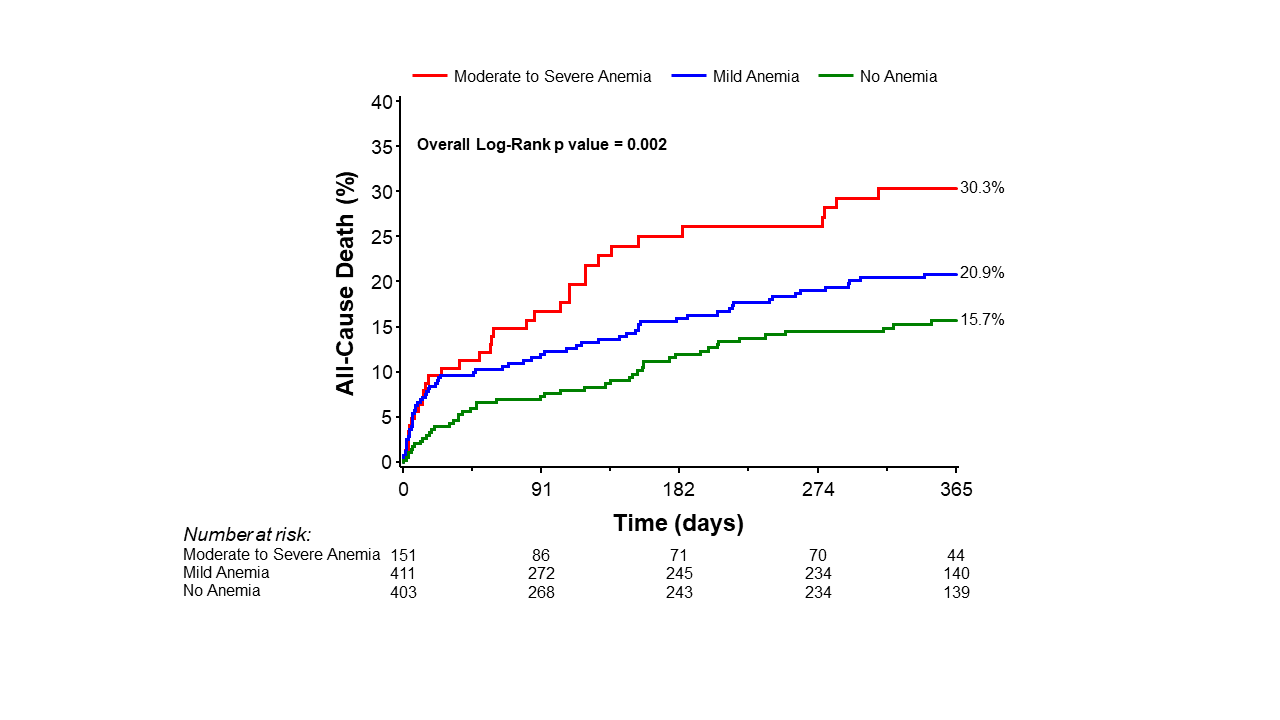


**Supplemental Figure S3. Forest Plot of the Adjusted Hazards Ratio for MACCE Rates at Estimated by Cox Proportional Hazards Regression Model excluding patients on dialysis.**

**Supplemental Figure S3A. Forest Plot of the Adjusted Hazards Ratio for MACCE Rates at 30 Days Estimated by Cox Proportional Hazards Regression Model excluding patients on dialysis.**

**
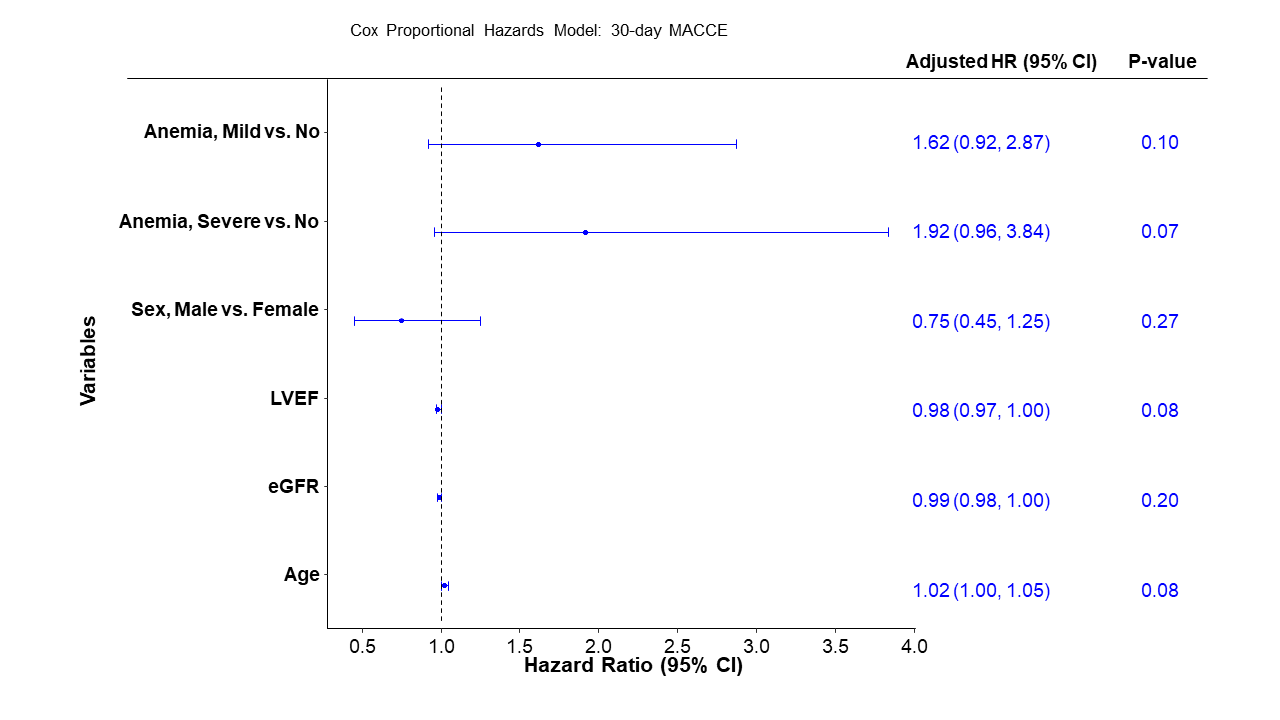
**

**Supplemental Figure S3B. Forest Plot of the Adjusted Hazards Ratio for MACCE Rates at 90 Days Estimated by Cox Proportional Hazards Regression Model excluding patients on dialysis.**

**
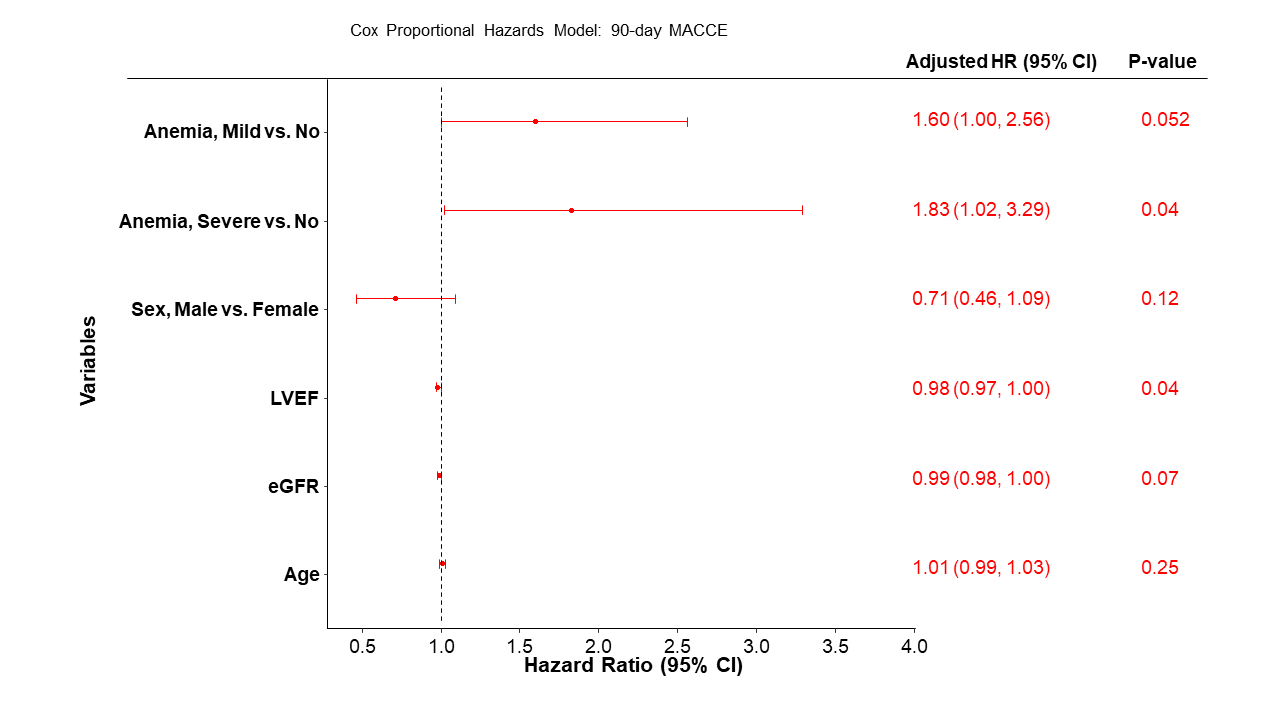
**
